# Naloxone dosing by non-medical first-responders at opioid overdoses: findings from a qualitative interview study

**DOI:** 10.1186/s12954-025-01203-1

**Published:** 2025-04-18

**Authors:** Joanne Neale, James Cassidy, Sarah Cosgrove, Ben Carter, Teodora Dascal, Clare Mackie, Nicola Metrebian, John Strang

**Affiliations:** 1 National Addiction Centre, Institute of Psychiatry, Psychology & Neuroscience, King’s College London, 4 Windsor Walk, London, SE5 8AF UK; 2 Centre for Social Research in Health, University of New South Wales Sydney, Sydney, NSW 2052 Australia; 3 South London & Maudsley (SLaM) NHS Foundation Trust, Maudsley Hospital, Denmark Hill, London, SE5 8AZ UK; 4 Biostatistics and Health Informatics, Institute of Psychiatry, Psychology & Neuroscience, King’s College London, Memory Lane, London, SE5 8AF UK

**Keywords:** Dosing, Naloxone, Training, Opioid, Overdose, Qualitative

## Abstract

**Background:**

Opioid-related deaths are increasing globally, and synthetic opioids intensify overdose risk. Naloxone can prevent fatalities, although too much can precipitate withdrawal and other negative reactions for the person overdosing. There is an increasing range of naloxone products, some providing very high doses, and this has generated different opinions on how much naloxone is necessary to save a life without causing harm. This paper explores how non-medical first-responders administer naloxone at opioid overdoses in the UK.

**Methods:**

Qualitative telephone interviews were conducted (2021–2023) with people who used services (*n* = 21, of whom 20 used opioids) and staff working with people who used opioids (*n* = 7). Participants had all been supplied with naloxone (predominantly injectable Prenoxad) and routine naloxone training as part of a separate cohort study. All had witnessed an overdose in the previous six months. Interviews were semi-structured, audio-recorded and transcribed. Data were coded and analyzed via Iterative Categorization.

**Results:**

Overdoses occurred within a framework of uncertainty. Participants were often unsure of the types and quantities of drugs consumed and did not always know if, or how much, naloxone had been administered. No deaths and few cases of withdrawal were reported, but other negative effects (including disorientation and anger) were common. On witnessing a potential overdose, participants made numerous decisions quickly. These included confirming the overdose and deciding whether naloxone was needed, who would administer it, when doses should be given, and when to stop dosing. These decisions were influenced by contextual factors, including the availability of a naloxone device, panic, prior knowledge of the person who overdosed, the helpfulness (or otherwise) of others present, and any training previously received.

**Conclusions:**

Naloxone dosing is complex and often reactive rather than purely scientific. Non-medical responders are competent at saving lives using naloxone, but do not always achieve resuscitation without negative effects. Findings highlight the value of offering optional advanced training and regular refresher training. This should focus on locally used naloxone products and dosing decision-making, plus experiential training that might enable people to anticipate how they would feel in a time-pressured overdose-related situation and so respond more calmly.

## Background

Over sixteen million people are affected by opioid use disorder (OUD) worldwide, with 140,558 people in treatment for OUD within the United Kingdom (UK) as of 2022 [1, 2]. The numbers of people using opioids and opioid-related mortalities are increasing in England and Wales, with 67.5% more fatal opioid-related overdoses occurring in 2022 (*n* = 2,261) than in 2012 (*n* = 1,350) [3]. A key factor driving the rise in opioid-related deaths globally is a change in the type of opioids being sold and used, with synthetic opioids (particularly fentanyl and its analogs, and also recently nitazenes) becoming more common [4, 5]. These synthetic opioids are significantly more potent than naturally occurring opioids, making their use a high-risk factor for overdosing [6]. In the UK, there is emergent evidence of synthetic opioids circulating with nitazines being more prevalent than fentanyl. Nitazines were detected in 179 fatal overdoses between 1st June 2023 and 31st May 2024 [7].

Naloxone is an opioid-antagonist that reverses the effects of opioids and can prevent potentially fatal respiratory depression [8, 9]. When administered to a person who has overdosed, the action is rapid (normally evident within a few minutes), but naloxone’s relatively short half-life (60–120 min) means that respiratory depression could reoccur [10, 11]. In addition, too much naloxone (‘over-antagonism’) can precipitate withdrawal in people who use opioids [12–14]. Opioid withdrawal can make people feel very unwell, restless and agitated, potentially decreasing their willingness to engage with medical professionals and increasing the likelihood that they may seek further substances to alleviate their discomfort [13, 15, 16]. Whilst nalmefene is also approved to reverse opioid overdoses in the United States (US) [16, 17], it is a stronger, longer-acting opioid antagonist than naloxone and is more likely to precipitate withdrawal, and for a longer period of time. Accordingly, naloxone remains the most widely used treatment for opioid overdoses internationally [16, 18].

The use of naloxone was originally restricted to medical and emergency personnel [19], but it is now widely provided to non-medically trained people who are likely to witness an overdose. This includes, amongst others, non-medical professionals (such as those who work in drug treatment or housing services), people who use opioids (both in and out of treatment), and the family, friends and peers of people who use opioids. People who use opioids can secure naloxone to administer to others if they witness an overdose or to give to family and friends to administer to them if they personally overdose. Although uncommon, it is additionally possible for people who use opioids to administer naloxone to themselves [20]. Whilst there is an increasing range of naloxone products [16], two have been available over recent years to community members in the UK [21]. These are Prenoxad and Nyxoid. Some people may prefer and use one device rather than the other [22], whereas others may own both products for use in different situations or in combination.

Prenoxad consists of a syringe pre-filled with 2 ml of naloxone (concentration = 1 mg/1mL) where the syringe contains five 0.4 mg doses [23, 24]. The syringe is marked with five black lines to help the person administering naloxone know when one dose has been given. Clinical guidelines indicate that a single dose (0.4 mg) should initially be injected intramuscularly, followed by a cycle of 30 chest compressions and two rescue breaths where possible. If the person overdosing remains unresponsive, another dose of Prenoxad should be administered, followed by three resuscitation cycles. This process should then be repeated until the person overdosing regains breathing/consciousness, at which point the injection, and any remaining naloxone solution, should be disposed of safely as per the accompanying instructions [25]. Nyxoid is a concentrated naloxone nasal spray that is provided in a twin-pack, with each spray containing one full naloxone dose (1.8 mg/0.1mL) which is administered into a single nostril. If the first dose does not result in recovery within the next 2–3 min, another full dose should be administered into the other nostril, with further additional doses administered as needed [26].

In the UK, drug treatment services have been able to distribute Prenoxad since 2015 and Nyxoid received similar licensing in 2019, soon after its arrival as a new medicinal product [27, 28]. Since then, both the accessibility and use of naloxone have increased, with 26% of people in treatment for OUD in England supplied with a naloxone kit in 2020 and 18.9% more overdoses being treated with naloxone in 2019 than in 2016 [29]. As the number of opioid-related deaths has continued to increase, the UK Government recently pledged to further expand the availability of naloxone across the community [30]. The pre-provision of naloxone to non-medically trained community members, along with training on supportive actions (such as how to recognise an opioid overdose, place the person overdosing in the recovery position, administer naloxone, give rescue breaths, and call for an ambulance) is popularly termed take-home naloxone (THN) [16, 31, 32].

THN programmes are associated with reduced opioid overdose mortalities [33–35], but the training component is not standardized, tends to be brief (up to 30 min), and is not always attended by people supplied with naloxone [36–39]. To improve this, many THN programmes have adopted train-the-trainer models whereby peer workers, who are often compensated financially for their time, are given in-depth training in overdose identification and appropriate response. They then provide THN kits and similar training to members of their communities [40–42]. This approach can improve the extent of community members’ knowledge of overdoses and how to respond, whilst simultaneously boosting the respect, self-esteem, and confidence of peer-trainers [37, 40, 41]. Recently, online naloxone training programmes have started to be developed, but these are variably available and not yet well studied [43].

Despite naloxone’s evident effectiveness, stigma, insufficient knowledge about the administration process, and misconceptions about its short- and long-term effects are known barriers to carriage and use [44–47]. In addition, people who have been treated with naloxone for an opioid overdose often experience unpleasant side effects, particularly if they have received large doses [48–50]. Alongside withdrawal symptoms (as described above), these negative effects can include disorientation and anger which have the potential to undermine the reputation of naloxone and further impede its uptake [13, 23, 51, 52]. Countering this, some first-responders (particularly people who themselves use opioids) titrate the naloxone dose to ensure the smallest amount required is administered and/or communicate empathetically with the person who has overdosed to minimise any distress [53–55].

The amount of naloxone that should be given at an opioid overdose has become a contested topic [16, 56]. Several very high-dose naloxone products, including a 5 mg/0.5mL intramuscular injection and a 7.2 mg/0.1mL intranasal spray, have recently been developed and approved in the US [9, 16]. Some commentators argue that these high doses are needed to reverse synthetic opioid overdoses [56, 57] and there is evidence that medical professionals use higher naloxone doses when faced with a presumed synthetic opioid overdose [58, 59]. Conversely, others contend that these new products are unnecessary. They question the evidence suggesting that synthetic opioid overdoses always require very large doses, note that opioid reversals using standard dose products continue to have a 99% success rate even in communities where fentanyl has been introduced, and maintain that people may be reluctant to administer high-dose products because of the increased likelihood of precipitating withdrawal [16, 22, 60, 61].

Using qualitative data from New York City, Parkin and colleagues explored how and why people who use drugs decide to administer a second dose of naloxone [55]. Findings revealed that people often acted outside the recommended dose response interval of 2–4 min and administration of the second dose was related to a range of factors including panic, perceived urgency, delays in retrieving naloxone, and the recipient’s response to the first naloxone dose. To-date, there has, however, been no qualitative analysis of how non-medical first-responders administer naloxone at opioid overdoses in the UK. Responding to this gap, the current paper draws upon qualitative interviews conducted with people who had recently witnessed a presumed opioid overdose to address three research questions: (1) How much naloxone is administered, by whom, and with what outcomes? (2) What decisions about naloxone administration, and particularly dosing, are made and why? and (3) What circumstantial factors influence naloxone administration and dosing decision-making? Findings are then discussed with reference to the existing literature on naloxone dosing before recommendations for THN programmes are suggested.

## Methods

Data were generated from a qualitative study nested within a large prospective cohort study of the effectiveness of naloxone administration and overdose reversal by community members [62]. The cohort study was designed to assess the rate of naloxone administration and subsequent reversal of opioid overdose by community members who witnessed an overdose to determine how closely real-life naloxone use conformed to overdose response training, and to obtain a better understanding of naloxone administration and overdose reversals by community members. The aim of the qualitative study was to add depth and detail to the main quantitative findings.

Participants were recruited to the prospective cohort from 22 drug treatment and harm reduction services in England, Scotland, Wales, and Sweden. They included people who used opioids (*n* = 1,030); non-medically trained professionals working with people who used opioids (*n* = 208); and family members, friends or a close contact of a person who used opioids (*n* = 169). All were supplied with a naloxone kit (if they did not already have one) and routine naloxone training (or refresher training) from their recruitment service at enrolment to the study. Quantitative data (to be reported separately) were collected between June 2021 and June 2024 from structured questionnaires completed at baseline and again if the participant witnessed an overdose within the next six months.

Qualitative telephone interviews were additionally conducted with a sub-group of participants who had witnessed a presumed opioid overdose in England, Scotland or Wales between September 2021 and July 2023. The main type of naloxone being distributed across the UK recruitment sites during this period was Prenoxad (injectable pre-filled syringe), with minimal distribution of Nyxoid (concentrated naloxone nasal spray). The contact details of eligible cohort participants who agreed to being approached for a qualitative interview (*n* = 73) were passed to a trained qualitative researcher who endeavoured to telephone them to describe the nested qualitative study. In total, 44/73 were contactable, of whom 33 agreed to participate and provided informed consent to conduct the qualitative interview. Five were, however, subsequently excluded from the present analyses: two had not witnessed the overdose they reported; two had multiple inconsistencies in their accounts making interpretation difficult; and one described the same overdose as an earlier participant.

Each interview was guided by a semi-structured topic guide that covered the participant’s demographic characteristics and personal circumstances; current substance use and treatment; recollection of naloxone training; accounts of overdoses experienced and witnessed since enrolment in the study; details of the most recent overdose witnessed whilst in the study; and anything else the participant thought was important. The topic guide was developed by three academic members of the research team (one of whom also has a clinical background) to address a series of aims and objectives that supplemented the main quantitative questions. When asking about the most recent overdose witnessed, the topic guide explored, *inter alia*, the circumstances surrounding the overdose, onset of the overdose, response to the overdose, naloxone carriage and use, naloxone administration, effectiveness of any naloxone administered, and outcome of the overdose. Interviews lasted between 19 and 57 min (mean = 38 min) and were audio recorded using an encrypted recording device. The topic guide was used flexibly, and the audio of each interview was reviewed within 48 h to check for relevance and quality. Team members discussed the content of the interviews throughout the data collection period but no amendments to the topic guide were needed.

Every participant was thanked with a £20 shopping voucher on completion of their interview. As the interviews were being conducted remotely by telephone and we did not want to limit participation to people who had bank details for credit transfer, we used vouchers rather than cash. All recordings were transcribed verbatim by a professional transcription service and the transcribed interview data were entered into the qualitative software package MAXQDA24 [63] in preparation for systematic coding. The process of coding the data occurred after all interviews had been completed and was undertaken by one member of the team in discussion with a second member of the team. Codes were developed *a priori* (‘deductive coding’) to address the qualitative study aims and objectives. Four of these codes were relevant to the current analyses: ‘Naloxone administration’; ‘Decision-making regarding naloxone administration’; ‘Effectiveness of naloxone administration’; and ‘Negative consequences of naloxone administration’.

All data indexed to the four codes of interest were extracted from MAXQDA24 and analysed following the principles of Iterative Categorization [64, 65]. This involved summarising every segment of data indexed to each code and annotating it with the identifier of the participant making the point (so that it was always possible to see which participant/s had made which point/s). The summarised data for each code were then reviewed, grouped, and regrouped under headings and subheadings; a process which enabled themes and patterns within the coded data to emerge. Two members of the research team who had previously analysed the wider dataset as part of an unpublished report for the study funder also cross-checked the findings against the earlier report and established that there were no omissions or missing contextual data. Finally, the findings from all four codes were organised under the three research questions addressed in this paper and a textual account was produced.

### Participants

Participants (*n* = 28) included eighteen identifying as male and ten identifying as female (see Table 1). Their ages ranged from 26 to 63 years (mean = 42 years) and most (*n* = 23) identified as White British. In total, twenty were people who used opioids, seven were staff working with people who used opioids, and one was homeless and living in a hostel but did not use opioids. All twenty of those who used opioids were currently receiving opioid replacement therapy and nine were also currently using heroin. Other current substance use included crack cocaine, cannabis and diazepam. Staff roles included drug worker in a treatment service (*n* = 2); senior support worker in a homeless charity (*n* = 2); manager of supported accommodation (*n* = 1); homeless charity team leader (*n* = 1); and supported accommodation officer in a residential service (*n* = 1). To facilitate comparisons between staff and non-staff participants, people who used opioids have been grouped with the person living in a hostel who did not use opioids under the heading ‘people who use services’.

**Table 1:** Participant characteristics (self-reported)

Characteristic	Person who uses services^1^ (*n* = 21)	Staff (*n* = 7)	Total (*n* = 28)
Sex			
*Male* *Female*	16 5	2 5	18 10
Age in years			
*Mean (range)*	41 (26–58)	42 (26–63)	42 (26–63)
Ethnicity			
*White British* *Black British* *Mixed* *Other*	18 2 0 1	5 0 1 1	23 2 1 2
Recruitment country			
*England* *Wales* *Scotland*	17 3 1	0 7 0	17 10 1
Recruitment site			
*Drug Treatment Service* *Harm Reduction Service*	17 4	0 7	17 11
Type of naloxone supplied with study enrolment			
*Prenoxad* *Nyxoid*	20 1	7 0	27 1

^1^ To facilitate comparisons between staff and non-staff participants, people who used opioids have been combined with the person living in a hostel who did not use opioids as ‘people who use services’

Seventeen participants were recruited from England, ten from Wales and one from Scotland. Most (*n* = 17) had been recruited to the study from drug treatment services, although all seven staff had been recruited from harm reduction services. One person who used services had been supplied with Nyxoid at study enrolment, whereas everyone else had been given Prenoxad. All participants should have received some form of localized naloxone training from their recruitment service when joining the study, but one person who used services stated that she had not had any training, and another said that he had only been asked a few questions rather than receiving any meaningful instruction. Other people who used services, and most staff, reported that they had received naloxone training on multiple occasions over the years.

## Findings

Participants were often uncertain about the types and quantities of drugs consumed by the people whose overdoses they had witnessed. Heroin (either smoked or injected) was presumed to have been taken at every event, often with crack cocaine. In addition, there were occasional reports that people had likely drunk alcohol or used other substances such as spice or benzodiazepines (the co-use of opioids and benzodiazepines is common within the UK but particularly in Scotland) [66]. A small number of participants also thought that fentanyl might have been mixed with the heroin consumed but there was no additional evidence to support this.

Lack of clarity regarding substances taken is a known common feature of real-life overdose events [59, 61, 67]. It therefore becomes part of the context in which naloxone dosing routinely occurs and often needs to be factored into dosing practices and decisions as an unknown. With this limitation acknowledged, we present findings that relate to our three research questions, using anonymised quotations to illustrate key points.

### How much naloxone is administered, by whom, and with what outcomes?

Twenty-one of the twenty-eight participants reported that naloxone had been administered at the overdose they witnessed. Five were unsure because they had not personally seen any naloxone being given and two others said that they were certain no naloxone had been used because there had been no naloxone present. Prenoxad was administered on twenty of the twenty-one occasions at which naloxone use was confirmed. The type of naloxone used on one occasion was not stated and there were no reports of Nyxoid being used on its own. Prenoxad was, however, given in combination with Nyxoid on one occasion when a staff member administered one spray of Nyxoid followed by an entire syringe of Prenoxad and then another spray of Nyxoid. The participant explained how this was standard practice for their service:

“We’ll give them the nasal spray first… If they don’t come round, we’ll give them the injection… I would say maybe three or four times they don’t come round and, by the advice then of paramedics, we can give them a further nasal spray… generally, they come round by then.” (Staff #2, Female).

Thirteen participants (eleven people who used services and two staff participants) described how they had personally administered the naloxone. In addition, eight participants reported that other people (staff working in hostels and services, a friend, an acquaintance, an unknown bystander, paramedics or a hostel nurse) had given the medication. Administration by others had occurred when the participant did not have naloxone with them, or if another person present had been first to react, or if the participant had passed their naloxone kit to someone else to use. In a few cases, more than one person had administered naloxone; for example, two staff members responding together or a person who used services followed by a paramedic:

“I’ve given him the full dose [syringe of Prenoxad]… I was about to give him another… We had two, two sets of naloxone there. I was gonna give him another dose, and then the ambulance people turned up… and they gave him another load… I think they gave him two loads even.” (Person who uses services #3, Female).

In describing how much naloxone had been administered, participants used a range of terms (‘doses’, ‘levels’, ‘loads’, ‘administrations’, ‘thirds’, ‘quarters’ and ‘calibrations’ etc.) interchangeably and were often unable to define what exactly they meant by each term. As a result, it was impossible to standardise and compare the amount of naloxone administered across the last overdoses witnessed (for example, five doses might have been given as one administration or as five administrations). Complicating this further, participants had not always seen how much naloxone had been administered when it was given by someone else.

With these caveats, the most common number of doses administered was reported to be two (i.e., 2/5 of a Prenoxad syringe or two 0.4 mg injectable doses), although ‘three doses’, ‘the entire syringe’, and ‘one dose’ were also described. Equally, there were some occasions when a very large amount of naloxone had been administered. For example, one participant described giving ‘eight doses’ over a number of administrations, whilst another said that she had injected four entire naloxone syringes (i.e., twenty 0.4 mg doses). When probed about this by the interviewer, the participant explained how she had repeated the following titration with four separate syringes:

“There’s five, five lines, and I went down to the one, waited two to three minutes, went down to the two, waited two to three minutes, went down to the three [and so on].” (Person who uses services #20, Female).

No participant reported that the last overdose they had witnessed had resulted in a death and only two participants stated that the person being treated with naloxone had displayed physical withdrawal symptoms. Specifically, one person who had been treated with naloxone was observed to be sweating and shaking and began to vomit after regaining consciousness whilst another was ‘twitching’. Despite this, people who had been treated with naloxone exhibited a variety of other negative reactions. These ranged from minor signs of confusion, shock, or disorientation (e.g., not knowing where they were there or what had happened) to more irritable and angry outbursts and, in one case, violence:

“He thought the police were coming for some reason. And he just wanted to run out the house. Slapped his girlfriend across the face.” (Person who uses services #18, Female).

Additionally, some participants reported that people who had overdosed had seemed embarrassed or been apologetic. One staff participant also described how a woman who had overdosed had started crying when she regained consciousness and found that her partner had simultaneously overdosed and was unresponsive. Other participants said that people who had been treated with naloxone were uncooperative and refused routine recovery checks or to go to the hospital with paramedics. Occasionally, their reactions had included shouting or swearing at staff and paramedics or physically pushing them away.

Analyses indicated no evident relationship between the amount of naloxone given and negative outcomes. For example, some participants recalled how people had reacted angrily after being treated with two Prenoxad doses, whilst others only exhibited confusion or disorientation after a much higher naloxone dose. There were, however, more reports of people responding angrily when naloxone had been administered by non-professionals (e.g., participants who used services or other bystanders) than by professionals (e.g., staff participants or paramedics).

### What decisions about naloxone administration, and particularly dosing, are made and why?

Prior to administering naloxone, participants first had to decide whether an opioid overdose was occurring. In considering this, they routinely referred to observing physical signs such as unresponsiveness, no or shallow breathing, no or changing colour, no or losing consciousness, snoring, and/or weak pulse. Some participants also commented that seeing drug paraphernalia or knowing that the person who had overdosed used drugs or had recently left prison helped them to decide it was an overdose. A few staff participants added that they tended to assume an overdose was occurring if someone was exhibiting overdose-like symptoms because of the high levels of substance use disorders experienced by their clients.

Participants frequently indicated that they had instinctively thought of naloxone once they had decided they were witnessing an overdose. Some followed this with a decision to administer naloxone because the person’s breathing was shallow or laboured, whilst two others determined that naloxone was needed because there was no breathing at all:

“I put her in the recovery position… But then obviously she started to snore… Like it sounded like snoring… I know that’s a sign of overdose. She went grey, her lips went blue, she stopped breathing, like completely stopped breathing. So, I administered one of the doses of naloxone.” (Person who uses service #20, Female).

One staff participant who worked in a hostel also described how she had first checked the oxygen levels of the person who had overdosed using a portable finger-tip pulse oximeter, had performed other observations, and had access to an oxygen tank. Meanwhile, other staff participants explained how they automatically administered naloxone when someone was displaying signs of overdose even if they were not certain that opioids had been consumed:

“We would use the naloxone anyway, you know, even if we didn’t know what it was.” (Staff #2, Female).

In determining where in the body to administer naloxone, participants generally opted for the most accessible large muscle. Accordingly, Prenoxad was most often injected into the thigh, although others referred to the arm, buttocks, or shoulder. Many participants described how they had injected naloxone through a layer of clothing whilst others had adjusted clothing or injected directly into the skin if a person was wearing a t-shirt or shorts. When Nyxoid was given with Prenoxad, the staff participant reported that each dose had been sprayed into alternate nostrils.

More than one dose of Prenoxad was administered in about half of the cases where naloxone was known to have been used. The main reason participants gave for administering additional doses was that the first had not revived the person (although sometimes the person’s breathing had improved, or they were more conscious, or their heart was beating a little faster). In two cases, the person had relapsed back into the overdose, so naloxone was readministered. A few participants also explained that they thought the person who had overdosed may have taken a combination of substances and/or the naloxone already given to them did not seem to be working so further doses were needed:

“He [another person in the house where the overdose occurred] gave him one dose, did nothing, two doses, did nothing. I said… We sort of agreed, I said ‘Get it all in there’, bosh, and then he come round.” (Person who uses services #1, Male).

Whilst some participants (both people who used services and staff) reported that they had waited between doses, others said that they had not waited any time at all. When participants had waited, times varied from seconds to minutes, with the most frequently identified time being a minute. Some participants were quite precise in reporting this timeframe (for example, saying it was two minutes or three minutes). Others commented on how difficult it was to remember the time between the doses, particularly given the anxiety they were experiencing during the situation:

“I couldn’t remember. It could have been… It felt like ages, but in them situations you don’t know.” (Person who uses services #8, Male).

The only reason participants gave for ceasing naloxone administration was that the person had regained consciousness.

### What circumstantial factors influence naloxone administration and dosing decision-making?

An initial factor affecting the decision to administer naloxone was whether a naloxone kit was available. At two overdoses, participants (and others present) did not have naloxone with them, so none was used. One participant who found himself in this situation was in the house of a person who had overdosed and explained:

“You’ve gotta deal with him [person who had overdosed] rather than run around the house looking for it [naloxone, and] maybe not finding it.” (Person who uses services #10, Male).

Sometimes participants who were not carrying naloxone asked others present or nearby for a naloxone kit, ran to a local service to find one, or called paramedics or treatment service or hostel staff. Whilst the decision to seek naloxone from elsewhere was often made quickly, the process of securing a kit inevitably delayed administration of the first dose. On other occasions, the participant and others present did not appear to consider naloxone at all or only discussed it after the event:

“Afterwards… walking back up the road and that, man, then it [naloxone] might have been mentioned, ‘Oh man, could have used that, man. I’ve just got this kit’… But at the time… then, no, never brought up in the conversation at that point.” (Person who uses services #19, Male).

Panic and a sense of urgency routinely influenced decisions about administering naloxone. For example, one participant stated that he had ‘snatched’ the device out of the hand of the partner of a person who had overdosed because she was fumbling, and they needed to act quickly. Other participants explained how panic and adrenalin had made it hard for them to remain calm, pause between administrations, or calculate the number of naloxone doses given. Reflecting this, one person who used services described how, in the heat of the moment, he had forgotten to administer the naloxone ‘in two halves’ and had, instead, administered it all at once.

For a small number of participants, decision-making was additionally undermined by not knowing what substance(s) a person had taken (particularly if reports of very strong heroin or fentanyl were circulating), how much of a substance they had consumed, or how long ago they had used substances. This had either made them unsure about whether they should administer naloxone at all or prompted them to administer a large dose quickly as a precaution:

“I thought, give him the whole lot. Because I don’t know how long… how much. I don’t know how much he had. He could have put two bags in or, I don’t know… So, I just thought give him the lot.” (Person who uses services #2, Male).

In contrast, other participants felt that they had been able to make an informed decision about naloxone dosing because others present had told them what substances had been taken or because they knew the person who had overdosed:

“As soon as I knew who they were [person who regularly consumed heroin], I knew straight away that it was gonna be a potential overdose.” (Staff #6, Female).

The presence of other people (virtually or physically) was a further factor influencing decision-making. Thus, some participants (mostly staff) explained how the emergency services had provided support by mobile phone throughout the overdose, including advice on giving naloxone. Meanwhile, people who witnessed overdoses in houses or public spaces sometimes coordinated a response even if they did not know each other. For example, one person might retrieve a naloxone kit, assemble it, move the person into a better position for administering naloxone, give rescue breaths, or call the emergency services whilst another proceeded with the injection.

Staff participants also often described how they responded to overdoses that occurred at work as a team. At these events, naloxone administration was sometimes discussed aloud whilst, on other occasions, staff said that they worked together instinctively in silence. Having successfully dealt with multiple overdoses together previously appeared to make decision-making easier. Furthermore, one staff participant emphasised how having confidence in her colleague made the decision to administer additional doses of naloxone very straightforward:

“He [colleague] has dealt with more overdoses than I have. So, he’s like, ‘You know, it’s been over three minutes. Shall I give him another go?’ I’m like, ‘Yeah, definitely, go for it’.” (Staff #1, Female).

Despite these examples of collaboration and team working, bystanders did not always contribute helpfully to decision-making. For example, some participants stated that other people present had panicked that the police would arrive and so had left quickly or were running about shouting and hindering the response. Additionally, two people who used services explained how others present had told them not to call an ambulance in case the police came whereas others said that they had not called an ambulance because it was not considered ‘the done thing’. A few participants also reported that they had had to ignore friends or associates of the person who had overdosed who were insisting that naloxone should not be given in case it put the person into withdrawal or ‘spoiled their buzz’.

Finally, decision-making was sometimes influenced by the training participants had or had not received. Thus, a few participants reported that they had decided that naloxone was needed after remembering the signs of overdose they had been taught, and others recalled that they were supposed to re-administer naloxone every two to three minutes if there was no response. One staff participant also stated that she had followed her service’s protocol to use Nyxoid first and then move to Prenoxad if needed. Meanwhile, others said that they had administered large doses of naloxone because they had been told by trainers that this would not do any harm:

“Obviously there’s gauges on… the syringe. But she [naloxone trainer] said, ‘To be honest with you just use the whole dose'… She said it’s not gonna hurt anybody.” (Staff #2, Female).

Notwithstanding the knowledge participants often had, a few people who used services and one staff participant said that they had felt scared or not known what to do when faced with a real overdose. Additionally, one staff participant said that he could not administer Nyxoid, even though the service where he worked used it, as he had not yet been trained. In response, several participants expressed a desire for additional or refresher training.

## Discussion

As long ago as 2005, administering naloxone was likened to ‘walking a prescribing tightrope’ between adequate resuscitation and precipitation of unpleasant withdrawal symptoms [68]. With the emergence of high potency synthetic opioids and new higher-dose naloxone products (as well as the longer-acting opioid antagonist nalmefene), determining the optimal naloxone dose has become contested [16]. This paper has considered how non-medical first-responders administered naloxone while responding to opioid overdoses in the UK. By addressing three research questions, findings have indicated that naloxone dosing is a complex and often reactive, rather than purely scientific, process. This is because it occurs within a framework of uncertainty, with multiple decisions needing to be made quickly, and complex contextual factors affecting administration.

The framework of uncertainty in which non-medical responders administer naloxone includes not knowing which substances (or combinations of substances) have been consumed, when or in what quantities. This is compounded because of changing drug markets [14, 56, 57], and because people often do not know the person who overdosed or their drug taking behaviours [59, 69]. Internationally, there is also an increasing range of naloxone products, with different formulations, concentrations, and delivery systems [9, 14]. This can make it hard for both medical and non-medical first-responders to assess how much naloxone is required and how much is being administered and absorbed, especially as the bioavailability of these products varies [9, 70].

Naloxone administration additionally requires numerous decisions to be made in a short timeframe. These decisions relate, *inter alia*, to whether the event being witnessed is an opioid overdose, whether naloxone is needed, and when the first and any subsequent doses should be administered. In a recent call for an opioid response standard of care, Russell and colleagues argued that naloxone should only be given if the person who has overdosed cannot be wakened, and their lips or fingertips have changed colour [16]. Dosing should then be repeated after three minutes if the person is not breathing at least once every five seconds. We found evidence that non-medical responders sometimes administered naloxone whilst people were still breathing, did not always titrate the dose, and encountered difficulties determining the intervals between administrations. Equally, negative outcomes (ranging from disorientation to anger) were common.

As previously described by Parkin and colleagues, dosing decisions were influenced by a range of circumstantial factors [55]. Alongside a perceived sense of urgency and panic, these included the availability of naloxone, whether other people present helped or hindered, whether paramedics were called, memory of any training received, and, for staff, workplace protocols. People who use opioids can be reluctant to call the emergency services in case the police arrive [36], and protocols to circumvent fear of arrest have been implemented in many places [71–73]. In the UK, police do not attend ambulance calls unless they are specifically summoned (e.g., because of violence or death). Nonetheless, our study showed that concerns about police attendance still impeded the provision of medical care. This highlights that non-attendance protocols need to be better publicized and implemented. Our data also indicated that overdose protocols and trainers sometimes recommend administering naloxone as soon as an overdose is suspected and/or giving multiple doses at once in the (mistaken) belief that naloxone can do no harm. Training and work-based practices need to be updated to ensure they are based on the best available evidence.

Despite these examples of suboptimal dosing, all overdoses described were successfully reversed. Moreover, participants generally seemed confident in identifying an overdose, determining that naloxone was needed, and administering the first dose. Some successfully titrated subsequent doses [53, 54] and there was limited evidence of precipitated physical withdrawals even though some gave very large quantities of naloxone. These findings confirm that non-medical first-responders can skillfully administer naloxone [53, 74]. People who used services were, however, more likely than staff to report an angry response from the person who overdosed. Research has suggested that withdrawal symptoms and anger after naloxone administration may in fact be unrelated, with anger (but not withdrawal) being less likely when the person administering naloxone has a positive or reassuring communication style [54]. As staff had often reversed multiple overdoses previously and frequently worked as a team according to a service protocol, their actions were possibly calmer and their communications clearer than someone responding to an overdose for the first time [23, 53, 54].

Overall, the analyses we present highlight the potential value of more advanced overdose training [73, 75]. Naloxone administration is only one component of managing an opioid overdose emergency. Other key activities include overdose identification, mobilizing support, following basic first aid instructions, and post-resuscitation management [16, 74]. It seems doubtful that all these topics, as well as the uncertainties and complexities of naloxone dosing, can be thoroughly discussed within brief THN training sessions lasting thirty minutes or less [38, 76]. Certainly, we must avoid creating barriers to THN uptake by requiring lengthy training that is burdensome and deters people from engaging. Nonetheless, it seems sensible to offer additional training, and to encourage participation in such additional training, so that those who are interested in learning more can respond with greater confidence and proficiency.

Calls for improved THN have been made previously [32, 37, 46]. Based on our findings, we suggest that THN providers offer training that covers locally used naloxone products and dosing decision-making, including when to initiate dosing, how to effectively titrate the dose, when to stop administering, and how to communicate calmly with the person overdosing during and after naloxone administration [23, 53]. This could be accompanied by more experiential training that includes simulating real-life overdose events and role play so that people acquire a sense of how they might feel in a panicked and time-pressured overdose-related situation. Hearing the first-hand accounts (in person or pre-recorded) of people who have responded to an overdose may also provide valuable new learning. Such methods have the potential to reinforce the importance of carrying naloxone whilst enabling people to feel more knowledgeable and prepared.

### Limitations

Limitations of our study include small sample size and the fact that all twenty participants who used opioids were receiving opioid replacement therapy, and most were recruited from drug treatment services. This restricts generalizability and the findings cannot necessarily be extrapolated to people not in treatment or using harm reduction services. Additionally, our findings relate almost exclusively to Prenoxad (which is designed with five doses in one syringe) and we have no reports of the experience of witnessing a fatal overdose. Whilst we identified some apparent differences in how people who use services and staff working in services respond, these require further study. Equally, it would be valuable to have data on how those who have personally been treated with naloxone perceive the relationship between the type and dose they received and how they felt afterwards. Despite these weaknesses, our analyses show consistencies with other international research which has, for example, found that most overdoses are treated with multiple naloxone doses [14, 77, 78], too much naloxone can produce negative outcomes [37, 39, 60], and non-medical responders understand the benefits of dose titration [53]. These similarities suggest that our findings have transferability to other naloxone products and settings.

## Conclusions

The aim of naloxone administration is to save life, but we should always seek to do this without causing distress or harm to the person who has overdosed [16]. Dosing is a complex practice involving multiple decisions that occur quickly in the context of many unknowns and circumstantial factors that cannot always be controlled. This makes it difficult, and potentially impossible, to give precise instructions on how much naloxone to give when encountering an overdose, particularly in a non-medical setting. Since we need non-medical first-responders to be as prepared as possible, it is important to offer them opportunities for advanced and regular refresher training, incorporating dosing decision-making and involving different learning approaches. We do not argue that this advanced training needs to be compulsory given that lives can be saved with only minimal instruction on naloxone use [32, 36, 37, 79]. However, our findings suggest that many people who attend, or work in, drug treatment services will welcome, and benefit from, the opportunity to learn more.

## Data Availability

The qualitative dataset generated and analyzed is not publicly available due to small sample size, sensitive data, and potential identification of organizations and individuals contra confidentiality agreements. Please contact the corresponding author for further information.

## Abbreviations

THN: Take Home Naloxone
UK: United Kingdom
US: United States
